# Inhibition of craniosynostosis and premature suture fusion in *Twist1* mutant mice with RNA nanoparticle gene therapy

**DOI:** 10.1126/sciadv.adx9763

**Published:** 2025-08-22

**Authors:** Samuel Swearson, Steve Eliason, Dan Su, Kevin G. Rice, Brad A. Amendt

**Affiliations:** ^1^ Carver College of Medicine, University of Iowa, Iowa City, IA 52242, USA.; ^2^ Craniofacial Anomalies Research Center, University of Iowa, Iowa City, IA, 52242.; ^3^ School of Medicine, Stanford University, Stanford, CA 94305, USA.; ^4^ College of Pharmacy, University of Iowa, Iowa City, IA 52242, USA.; ^5^ College of Dentistry, University of Iowa, Iowa City, IA 52242, USA.

## Abstract

Craniosynostosis is a common birth defect affecting 1 of the 2200 live births causing severe skull and cognitive defects, due to premature cranial suture fusion. The current surgical treatments require invasive calvaria vault remodeling and cranial bone resection in the baby. We demonstrate that inhibition of *miR-200a* in *PMIS–miR-200a* mice results in coronal suture fusion (craniosynostosis). Therefore, we use overexpression of *miR-200a* to prevent suture fusion in *Twist1* mutant mice, a well-known model for craniosynostosis. We developed a PEGylated-peptide nanoparticle system to deliver plasmid DNA expressing *miR-200a* directly to the sutures of postnatal day 4 (P4) *Twist1* mutant mice before suture fusion. Injection of the *miR-200a* nanoparticles under the scalp before suture fusion at P7 to P10 inhibited suture fusion. Treatments increased Gli1- and Six2-positive suture stem cells and the thickness of the periosteum layer. The treated *Twist1^+/−^* mice increased body weight and were alert and active. We demonstrate an effective noninvasive gene therapy treatment for craniosynostosis.

## Teaser

A therapeutic approach to inhibit premature suture fusion in craniosynostosis without invasive surgeries can improve child health.

## INTRODUCTION

Most cases of craniosynostosis are isolated or nonsyndromic. Syndromic craniosynostosis accounts for 9 to 15% of all craniosynostoses, presents with associated features of the face, trunk, and extremities; and varies in severity and etiology (*1*, *2*). Early diagnosis and treatment are important because cosmetic and functional problems arise when the brain continues to grow while skull growth is restricted because of premature fusion. In normal development, sutures between cranial bones remain patent to house the rapidly growing brain, which doubles in the first six months of life and triples by the first year to reach two-thirds of the adult volume. In patients with craniosynostosis, early fusion of cranial bones leads to compensatory growth in other areas of the skull, resulting in abnormal head shape, which is apparent between the last trimester of pregnancy and the end of the first year of life (*3*, *4*). In more severe cases, affected patients may have increased intracranial pressure (ICP) and experience functional problems (such as breathing difficulty, choking or vomiting on feeding, relatively protruded and unprotected eyes, irritability, and developmental delays) and even death (*5*). Commonly performed surgical techniques include fronto-orbital advancement, open cranial vault remodeling, extended strip craniectomy, spring-assisted cranial expansion, and cranial vault distraction. Although techniques for initial cranial vault expansion and reshaping depend on the location and extent of deformity, variability in surgical practice patterns and surgeon experience has been reported in a recent national survey of craniofacial surgeons in the United States (*6*). Morbidity and complication rates also vary widely across different centers, ranging from 10 to 39% (*7*–*11*). Thus, a noninvasive, effective treatment to keep the cranial sutures open in patients with genetic anomalies would be a major advancement for better patient outcomes.

More than 40 genes have been found to underlie syndromic craniosynostosis. These genes are clustered in developmental signaling pathways including *BMP* and *Wnt* (*4*, *12*–*18*). Clinical presentations are variable among affected individuals even in syndromes caused by recurrent mutations (Apert, Muenke, and Pfeiffer syndromes), demonstrating the variable expressivity of these mutations (*4*). *Twist1* functions to coordinate the activities of the BMP and RTK pathways, and, in humans, *TWIST1* haploinsufficiency causes Saethre-Chotzen syndrome (*19*–*21*). These affected individuals exhibit coronal suture synostosis, and similar phenotypes are observed in mice (*16*, *22*). The *Mesp1^Cre^/Twist1^+/f^* heterozygous mice caused the neural crest cells to become ectopically located in the coronal suture and mesodermal cells to cross into the frontal bone region. This causes a loss of osteogenic-nonosteogenic boundary integrity in the *Twist1* mutant mice (*16*, *22*, *23*). However, *Twist1* loss of function in both lineages (neural crest and mesoderm) causes craniosynostosis.

We have previously shown that the bone morphogenetic and β-catenin/Wnt pathways are regulated by the *miR-200* family in cell cultures (*24*–*31*). Other reports show that the *miR-200* family can inhibit the expression of *Dlx5* and *TGF-b2/Smad* pathways required for osteogenesis and bone formation (*32*–*35*). To demonstrate the role and potential therapeutic use of *miR-200a*, we have made transgenic mice inhibiting *miR-200a* (*PMIS–miR-200a*). We have shown that inhibition of *miR-200a* increases cranial bone density (BD) and bone volume/tissue volume (*28*). Therefore, here, we demonstrate that the delivery of plasmid DNA overexpressing *miR-200a* packaged in PEGylated-peptide nanoparticles can effectively inhibit suture fusion in *Twist1^+/−^* mice.

## RESULTS

### Inhibition of *miR-200a* in mice results in suture fusion

**Fig. 1.**
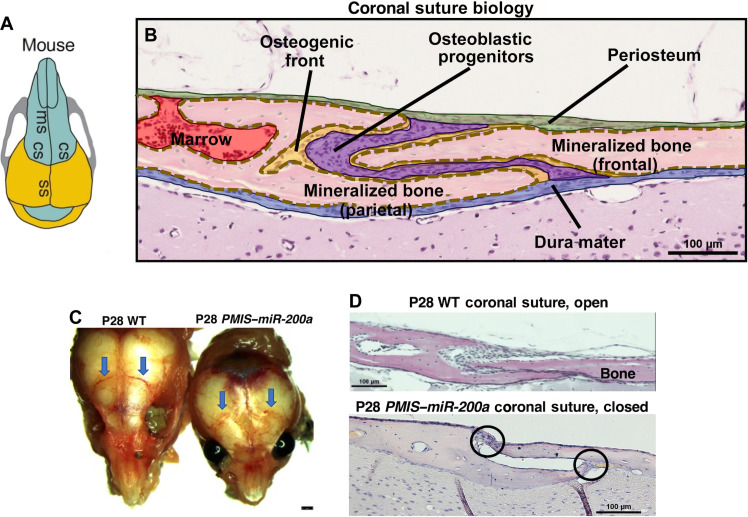
Coronal suture biology and suture fusion in *PMIS–miR-200a* mice. (**A**) Mouse skull (dorsal view) showing the metopic suture (ms), coronal suture (cs), and sagittal suture (ss). (**B**) Coronal suture biology diagram, showing the periosteum (green, layer covering the skull, dorsal most layer), mineralized bone (pink) and osteogenic fronts (yellow), osteoblastic progenitors or suture cells (purple), and dura mater (blue, layer between the brain and skull). (**C**) Dorsal view of P28 WT and *PMIS–miR-200a* mouse calvarium. The blue arrows denote the fused coronal sutures in the *PMIS–miR-200a* mice compared to unfused sutures in the WT mice. (**D**) Normal coronal suture biology is shown in P28 WT mouse sagittal sections, and fused sutures (black ovals) in the P28 *PMIS–miR-200a* mice.

We have previously shown that inhibition of *miR-200a* (*PMIS–miR-200a*) in a mouse model can cause increased craniofacial BD (*28*, *31*). The coronal suture biology is shown in (Fig. 1, A and B). Between the two calvaria bone forming regions are osteoblastic progenitors that contain suture stem cells. These cells contribute to the osteogenic fronts of the overlapping frontal and parietal bones. Progenitor cells are also localized in the periosteum and dura mater layers (Fig. 1B). The fusion of the coronal suture is 100% penetrant in the *PMIS–miR-200a* mice (*n* = 18). The postnatal day 28 (P28) *PMIS–miR-200a* mice have small heads and fused coronal sutures compared to wild-type (WT) mice (Fig. 1C). Analyses of P21 *PMIS–miR-200a* head size shows that the nasal bone and parietal bone lengths are decreased significantly compared to those of WT mice (fig. S1). Therefore, the total length of the *PMIS–miR-200a* heads are shorter, due to the effect of coronal suture fusion (*n* = 4, *P* < 0.01). Calvaria sections of P28 *PMIS–miR-200a* mice show fusion of the coronal suture and the abnormal head size and shape, typical of craniosynostosis phenotypes (Fig. 1D). These data show a role for *miR-200a* in regulating suture development. Thus, we hypothesized that overexpressing *miR-200a* would inhibit suture fusion in *Twist1^+/−^* mutant mice. We generated a *miR-200a* expression plasmid to test its effectiveness in preventing suture fusion in the *Twist1^+/−^* mouse model for craniosynostosis. To test our hypotheses, we first designed a nanoparticle system to deliver the *miR-200a* plasmid DNA to the sutures of *Twist1^+/−^* mutant mice before suture fusion.

### PEGylated-peptide nanoparticle formulation to package plasmid DNA

Previous studies had determined that a polyethylene glycol (PEG)–peptide DNA nanoparticle provided potent gene expression in vivo when injected systemically (*36*, *37*). The optimized polyacridine PEG-peptide bind tightly to plasmid DNA through poly-intercalation of Lys (Acr) residues and ionic binding of Lys residues (fig. S2A). Despite the overall positive charge, PEGylated DNA nanoparticles resist albumin binding when Lys (Acr) occupies position 17 (fig. S2B, PEG-peptides 1 and 2) (*38*). However, we altered the location of Lys^17^ (Acr) and Lys^16^, which result in nanoparticles that support protein binding (*38*) (PEG-peptides 3 and 4). The N-terminal Cys^18^ is modified with a reducible disulfide-linked to a 5-kDa PEG to allow PEG shedding (PEG-peptides 1 and 3). Substitution with a maleimide-linked PEG increases the stability of the PEG-peptide DNA particle (PEG-peptides 3 and 4). Plasmid DNA was prepared using either commercially available lysis and column purification kits or alkaline lysis and double-banded CsCl purification (*30*) and tested them for nanoparticle formation. Plasmid DNA from both preparations was suspended in either 1 mM NaCl or 1 mM CsCl and tested for nanoparticle size (fig. S2C). Dynamic light scattering measurements showed that, using CsCl–double-banded plasmid DNA, suspended in either NaCl or CsCl, the nanoparticles were ~95 nm (fig. S2C). However, plasmid DNA prepared using standard kit column purification yielded nanoparticle sizes from 115 to 125 nm (fig. S2C). The CsCl-prepared plasmid DNA was used in the experiments, as the smaller nanoparticle size allows for more efficient cell uptake (*39*). These data demonstrate that ultrapure plasmid DNA is packaged into small nanoparticles.

### Efficient delivery of plasmid DNA to cranial sutures and *miR-200a*–GFP expression

**Fig. 2.**
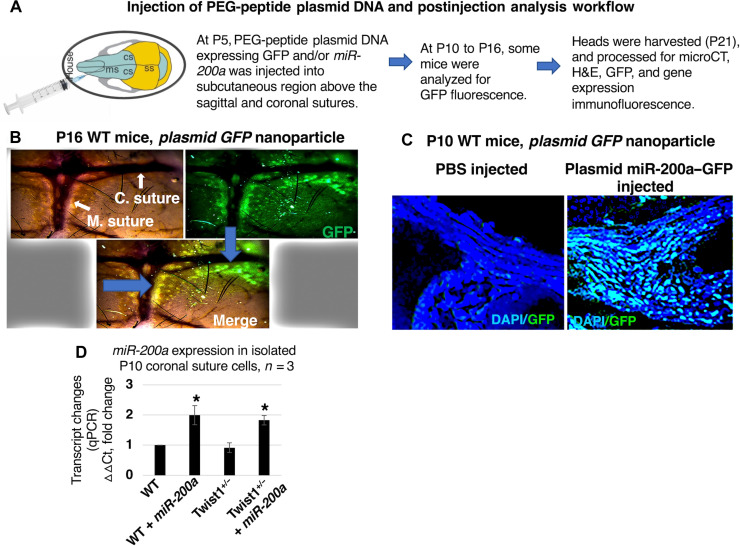
Delivery and expression of plasmid DNA expressing *pre-miR-200a*. (**A**) Procedure for injecting the PEG-peptide plasmid DNA expressing *pre-miR-200a*, under the scalp of mice. microCT, micro–computed tomography. (**B**) WT mice were injected at P4 and harvested at P16, the scalp was removed, and GFP fluorescence was visualized in the metopic (M.) and coronal (C.) sutures. (**C**) WT mice were injected at P4 with either PBS (control) or plasmid *miR-200a–GFP* and harvested at P10, and heads were fixed and processed for 4′,6-diamidino-2-phenylindole (DAPI) and GFP staining. The left panel is PBS controls showing DAPI staining but no GFP. The right panel is plasmid *miR-200a–GFP* treatment. (**D**) *miR-200a* expression in isolated P10 coronal suture cells from WT and *Twist1^+/−^* mice with and without *miR-200a* treatment. Transcripts were measured in three independent mice; fold change (*n* = 3, *P < 0.05).

To determine whether our nanoparticle system could transduce suture cells, we did pilot injections of WT mice with our PEG-peptide DNA. Our experimental design and timeline for injection and harvest to detect green fluorescent protein (GFP) expressed from the plasmid DNA are shown in Fig. 2A. Fluorescence microscopy and immunofluorescence (IF) techniques were used to determine the ability of the PEG-peptide DNA nanoparticles to transduce cells. Several concentrations of plasmid DNA expressing GFP were tested, and 5 to 10 μg of plasmid DNA was the lowest dose that reproducibly yielded strong GFP expression in the sutures (Fig. 2, B and C). GFP expression was observed 11 days after injection in the coronal and posterior frontal sutures in WT mouse calvaria (Fig. 2B). Sagittal sections of the cranial bone show GFP expression in the region of the open suture in the PEG/plasmid DNA *miR-200a*–GFP–injected animals, but not the control animals without *miR-200a*–GFP (Fig. 2C). To confirm *miR-200a* expression, suture cells were isolated from WT and *Twist1^+/−^* mice with and without *miR-200a* treatment (Fig. 2D). *miR-200a* expression was increased after treatments and, furthermore, the decrease in *Twist1* expression in *Twist1^+/−^* mice did not affect endogenous *miR-200a* expression (Fig. 2D). Peptide 1 was identified as the optimal nanoparticle to efficiently enter cells and express *miR-200a* from the CsCl–double-banded plasmid (fig. S2B). The other PEG-peptide nanoparticles were not efficiently taken up by the suture cells and/or promoted expression of the *pre-miR-200a* in the mice. They do work for other applications including systemic administration (*38*).

### *Twist1^+/−^* craniosynostosis mouse model and therapeutic inhibition of suture fusion

**Fig. 3.**
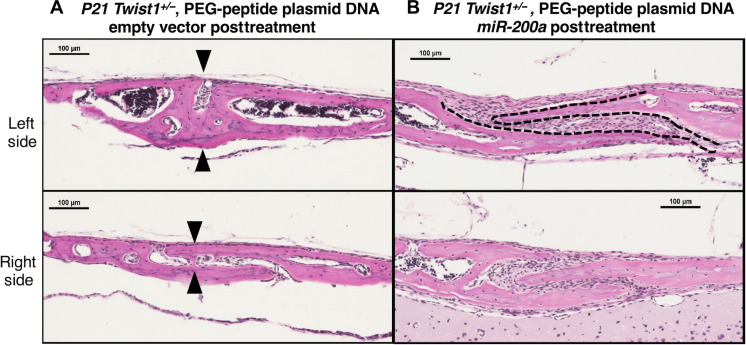
*Twist1^+/−^* suture patency was restored with *miR-200a* expression. (**A**) Representative H&E-stained images (left and right sides) of P21 *Twist1^+/−^* coronal sutures with EV treatments showing fused sutures, denoted by arrowheads. (**B**) Representative H&E-stained images (left and right sides) of P21 *Twist1^+/−^* coronal sutures with PEG-peptide plasmid *miR-200a* treatments showing open unfused sutures. The dashed lines separate the bone from the suture cells, and the arrowheads show fused bone.

**Fig. 4.**
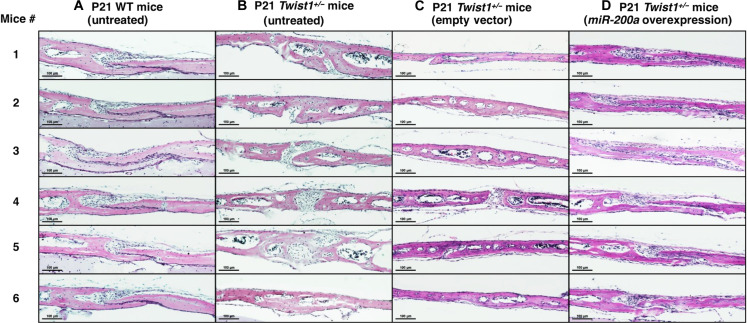
Suture fusion was inhibited in all *Twist1^+/−^* mice by treatment with PEG-peptide plasmid *miR-200a*. (**A**) P21 WT mouse coronal sutures were sectioned and H&E stained to visualize the frontal and parietal bones and suture cells. The WT mouse coronal sutures are all patent (open and unfused). (**B**) Coronal suture fusion in untreated P21 *Twist1^+/−^* mice; note that some sections do not show fusion; however, other sections show fusion; thus, the entire suture is not completely fused. (**C**) Coronal suture fusion in EV-treated *Twist1^+/−^* mice; again, in these sections, the suture is fused. (**D**) Coronal suture patency is restored in all sections of *Twist1^+/−^* mice treated with PEG-peptide plasmid *miR-200a*, similar to WT mice.

The *Twist1* heterozygous (*Twist1^+/−^*) mice provide an excellent model of craniosynostosis. *TWIST1* heterozygous loss of function mutations cause Saethre-Chotzen syndrome (*18*–*21*, *40*–*44*). These affected individuals present with coronal suture synostosis, and, in mice, a loss of function of a single *Twist1* allele exhibits a similar suture phenotype (*16*, *22*). We have found that 100% of the *Twist1^+/−^* and 0% of the WT littermates have craniosynostosis, but not complete coronal suture fusion (fig. S3). Because previous reports show that coronal suture fusion in *Twist1^+/−^* mice occurs between P9 and P13 (*23*), we choose to inject the nanoparticle *miR-200a* or control DNA at P4 to facilitate an early intervention. The initial experiments showed after injecting either empty vector (EV) nanoparticle DNA (5 μg) or nanoparticle m*iR-200a* plasmid DNA (5 μg) that *Twist1^+/−^* suture patency was restored with *miR-200a* expression at P21 (Fig. 3). Thus, one application of PEG-peptide plasmid *miR-200a* overexpression inhibits suture fusion 17 days posttreatment in a mutant genetic background of *Twist1^+/−^*. We next performed a series of experiments with *Twist1^+/+^* (WT) and *Twist1^+/−^* mutant mice treated with either nanoparticle EV DNA or nanoparticle *miR-200* DNA. It has been reported that the craniosynostosis phenotype is not completely penetrant (*23*); therefore, we analyzed six different treated and untreated mice for coronal suture fusion (Fig. 4). The P21 *Twist1^+/−^* mice had premature coronal suture fusion and 100% of suture fusion was inhibited by application of PEG-peptide *miR-200a* plasmid DNA (Fig. 4).

Additional analyses of P21 *Twist1^+/−^* mice (EV treatment, *n* = 8) with six sagittal sections laterally across the coronal sutures identify coronal suture regions that are both fused (see arrowheads) and open (fig. S4). In P21 *Twist1^+/−^* mice, the coronal suture is not continuously fused, only specific regions of the suture are fused causing craniosynostosis. All eight EV-treated *Twist1^+/−^* mice showed fused coronal sutures (fig. S4). With this knowledge, we analyzed p21 *Twist1^+/−^* mice (PEG-peptide *miR-200a* plasmid DNA treatment, *n* = 8) with six sagittal sections of the coronal sutures and found that all sutures were open, with no fused regions of the suture (fig. S5). All eight *miR-200a*–treated *Twist1^+/−^* mice showed open sutures demonstrating the efficacy of the PEG-peptide *miR-200a* plasmid DNA nanoparticle treatment. Again, after one treatment, the sutures remained patent after 17 days.

### Conditional inactivation of *Twist1* expression causes craniosynostosis

We performed experiments identical to those described for the *Twist1^+/−^* mice in a cell-autonomous mouse model, using the *Mesp1^Cre^* to conditionally inactivate *Twist1* in *Mesp1*-positive cells using the *Twist1*-floxed mice (*16*, *45*). The *Mesp1^Cre^/Twist1^F/F^* mice have premature coronal suture fusion through endochondral bone formation (*16*, *45*). The *Mesp1^Cre^ /Twist1*^*+/F*L^ mice also show premature suture closure with 100% penetrance. PEG-peptide *miR-200a* plasmid DNA was applied to the *Mesp1^Cre^/Twist1^+/FL^* mice in experiments identical to those described above, and we found that treatment with *miR-200a* prevented premature suture closure versus EV treatment by micro–computed tomography analyses (fig. S6, A to C; *n* = 3, three sections each mouse). Sagittal sections of these mice show suture fusion in the EV control–treated mice (fig. S6D); however, the coronal sutures remain open in the PEG-peptide *miR-200a* plasmid DNA–treated *Twist1* conditional-inactivated mice [fig. S6E, suture cells (SC)]. Using the *Mesp1^Cre^/Twist1^+/F^* mice, which generate suture closure through endochondral ossification, we show that *miR-200a* treatments are effective in both intramembranous and endochondral ossification processes (*23*, *45*–*49*). In two different mouse models of craniosynostosis, the *Twist1^+/−^* heterozygous knockout (KO) and *Mesp1^Cre^/Twist1^+/F^* conditional heterozygous KO mice, we demonstrated that *miR-200a* was able to inhibit the premature suture closure and prevent craniosynostosis in these mice.

### *miR-200a* treatment restores body size and weight in *Twist1* mutant mice

*TWIST1* haploinsufficiency is associated with craniosynostosis in patients with Saethre-Chotzen syndrome (*50*, *51*). Syndromic craniosynostosis is associated with neurocognitive dysfunctions and neuroanatomical changes, in addition to associated growth defects due to other aspects of gene function (*17*, *52*). To understand potential growth defects in *Twist1^+/−^* mice, all mice were weighed at the time of injection (P4) and at the time of euthanasia (P21). We noted that untreated *Twist1^+/−^* mice were smaller at P21 compared to WT littermates (fig. S7A). While craniosynostosis does not appear to affect body size in humans, mouse models do show reduced body size. Treatment of *Twist1^+/−^* mice with *miR-200a* nanoparticles restores their body weight to equal WT (fig. S7, A and B). This significant increase in weight occurs during early development, most likely due to the fact that the sutures remain patent and the lack of neurological changes. These results may also show a link between head-first development and body growth.

### *miR-200a* treatment increases the periosteum thickness, but not the dura mater, in *Twist1^+/−^* mice

**Fig. 5.**
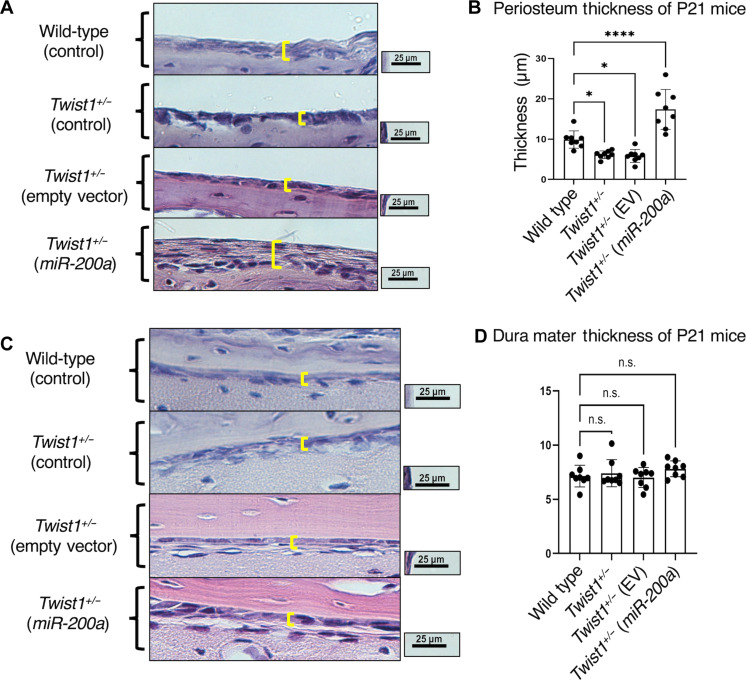
The periosteum layer increases thickness after PEG-peptide plasmid *miR-200a* treatments. (**A**) H&E-stained sections of P21 WT and untreated and treated *Twist1^+/−^* mice. The yellow brackets outline the periosteum layer, which is thinner in *Twist1^+/−^* control and EV-treated mice compared to that in WT. However, in *miR-200a*–treated *Twist1^+/−^* mice, the periosteum layer is thicker compared to all mice. (**B**) Quantitation of periosteum thickness (eight mice were analyzed for each treatment group, *n* = 8, *P < 0.05 and ****P < 0.0001). (**C**) H&E-stained sections of P21 WT and untreated and treated *Twist1^+/−^* mice. The yellow brackets outline the dura mater layer, which is unchanged in *Twist1^+/−^* control, EV-treated, and *miR-200a*–treated mice compared to that in WT. (**D**) Quantitation of dura mater thickness. *n* = 8 (eight mice were analyzed for each treatment group); n.s., not statistically significant.

To understand a potential mechanism for the inhibition of suture fusion in the treated *Twist1^+/−^* mice, we measured the thickness of the overlying periosteum layer and the underlying dura mater layer in the mice. We hypothesized that direct injection of nanoparticle *miR-200a* DNA under the scalp above the sutures may activate progenitor cells in the periosteum. The thickness of the periosteum in P21 *Twist1^+/−^* and *Twist1^+/−^* mice treated with EV was decreased compared to that in WT mice (Fig. 5, A and B). In contrast, the periosteum layer of *Twist1^+/−^* mice treated with *miR-200a* was significantly thicker compared to that of WT mice (Fig. 5, A and B). This expansion of the periosteum layer, which contains progenitor cells suggests that *miR-200a* is activating these cells. The thickness of the dura mater was unchanged between WT, *Twist1^+/−^*. and *Twist1^+/−^* treated with EV or *miR-200a* (Fig. 5, C and D). In addition, the total number of suture cells [4′,6-diamidino-2-phenylindole (DAPI)] was calculated in WT, *Twist1^+/−^*, *Twist1^+/−^* EV, and *miR-200a*–treated mice. This was done to determine whether *miR-200a* treatment replenished cells in the sutures. The *Twist1^+/−^ miR-200a*–treated mice contained more suture cells than *Twist1^+/−^* and *Twist1^+/−^* EV–treated mice (*n* = 3, *P* < 0.05) (fig. S8). There was no significant difference between WT and *Twist1^+/−^ miR-200a*–treated mice (fig. S8). Further experiments are required to understand whether these cells originate from the periosteum layer in the *miR-200a*–treated mice.

### Twist1- and Runx2-positive cells are restored after *miR-200a* treatment in *Twist1^+/−^* mice

**Fig. 6.**
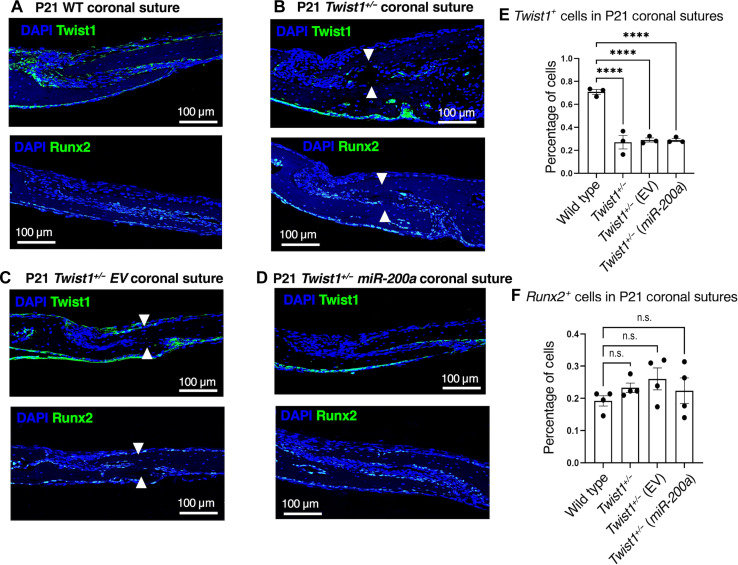
Twist1- and Runx2-positive cells are not affected after *miR-200a* treatment in *Twist1^+/−^* mice. (**A**) Twist1 and Runx2 IF expression in P21 WT mouse sagittal sections of the coronal suture. Twist1 (green staining) and Runx2 (green staining) are expressed in the suture cells and dura mater. (**B**) Twist1 and Runx2 IF expression in P21 *Twist1^+/−^* heterozygous mice, sagittal sections of the coronal suture. (**C**) Twist1 and Runx2 IF expression in P21 *Twist1^+/−^* heterozygous mice treated with EV nanoparticles, sagittal sections of the coronal suture. (**D**) Twist1 and Runx2 IF expression in P21 *Twist1^+/−^* heterozygous mice treated with *miR-200a* nanoparticles, sagittal sections of the coronal suture. (**E** and **F**) Quantitation of Twist1 (*n* = 3, three mice were analyzed for Twist1 expression) and Runx2 (*n* = 4, four mice were analyzed for Runx2 expression) expression levels in the mouse sagittal sections. ****P < 0.0001; n.s., not statistically significant.

To determine the molecular identity of cells, present in the *miR-200a*–treated *Twist1^+/−^* suture compared to that in *Twist1^+/−^* untreated or EV treated, we performed IF staining for Twist1, Runx2, Gli1, and Six2. We found that Twist1 expression was reduced in *Twist1^+/−^*-haploinsufficient mice as expected (Fig. 6, B and E) and decreased in the *Twist1^+/−^* EV treatment group (Fig. 6, C and E). However, this reduction was not corrected with *miR-200a* treatment (Fig. 6, D and E). Thus, *miR-200a* treatment did not restore Twist1 expression to WT levels as a mechanism to reverse suture fusion.

Runx2 is a known transcription factor involved in bone formation and regulates cranial suture closure by activating Fgf, Wnt, Hedgehog, and PthIh pathways in suture mesenchyme cells (*53*). Analyses of Runx2 expression in the *Twist1^+/−^* untreated and EV-treated mice show similar Runx2 expression levels as that in WT mice (Fig. 6, A to C and F). Runx2 expression is not changed in the *Twist1^+/−^ miR-200a*–treated mice compared to that in *Twist1^+/−^* untreated and EV-treated mice (Fig. 6, B to D and F). Therefore, the restoration of suture patency by *miR-200a* does not appear to be Runx2 dependent.

### Gli1- and Six2-positive suture cells are regulated by *miR-200a* treatment in *Twist1^+/−^* mice

**Fig. 7.**
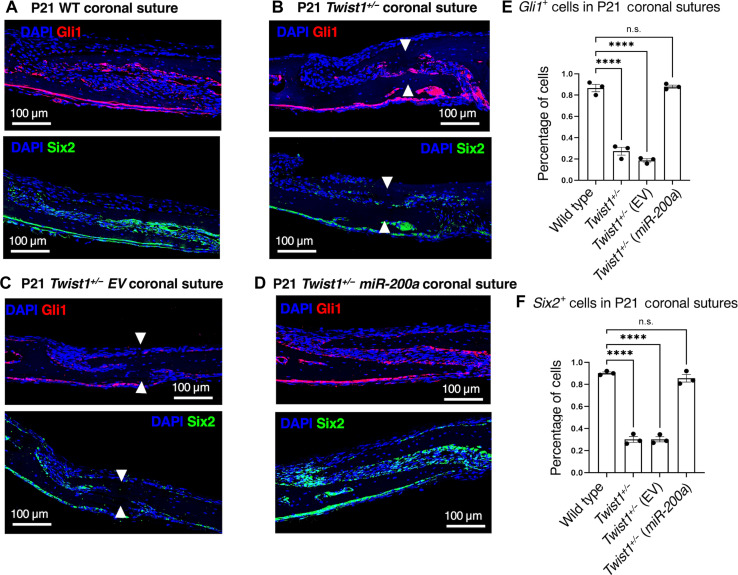
Gli1- and Six2-positive cells are regulated by *miR-200a* treatment in *Twist1^+/−^* mice. (**A**) Gli1 and Six2 IF expression in P21 WT mouse sagittal sections of the coronal suture. Gli1 (red staining) and Six2 (green staining) are expressed in suture cells and dura mater. (**B**) Gli1 and Six2 IF expression in P21 *Twist1^+/−^* heterozygous mice, sagittal sections of the coronal suture. (**C**) Gli1 and Six2 IF expression in P21 *Twist1^+/−^* heterozygous mice treated with EV nanoparticles, sagittal sections of the coronal suture. (**D**) Gli1 and Six2 IF expression in P21 *Twist1^+/−^* heterozygous mice treated with *miR-200a* nanoparticles, sagittal sections of the coronal suture. (**E** and **F**) Quantitation of Gli1 and Six2 expression levels in the mouse sagittal sections (*n* = 3, three mice were analyzed for Gli1 and Six2 expression). ****P < 0.0001.

Because Gli1 and Six2 are reported markers of suture progenitor cells, we analyzed WT and treated and untreated *Twist1^+/−^* mice for their expression (*47*, *52*). Both Gli1- and Six2-positive cells are expressed in the P21 WT coronal suture, in mesenchymal cells of the suture as well as the dura mater (Fig. 7A). Gli1^+^ and Six2^+^ suture cells are decreased in the *Twist1^+/−^* untreated and EV-treated mice (Fig. 7, B, C, E, and F). Gli1^+^ and Six2^+^ cells are restored to normal levels in *Twist1^+/−^ miR-200a*–treated mice as compared to those in WT (Fig. 7, A and D to F). Thus, *miR-200a* appears to activate these cells in the *Twist1^+/−^* mutant coronal suture. *miR-200a* restoration of Gli1^+^ and Six2^+^ cells suggests a potential mechanism for these cells to maintain suture patency after *miR-200a* treatment.

### Wnt10b and β-catenin are decreased in *miR-200a*–treated *Twist1^+/−^* mouse coronal sutures

**Fig. 8.**
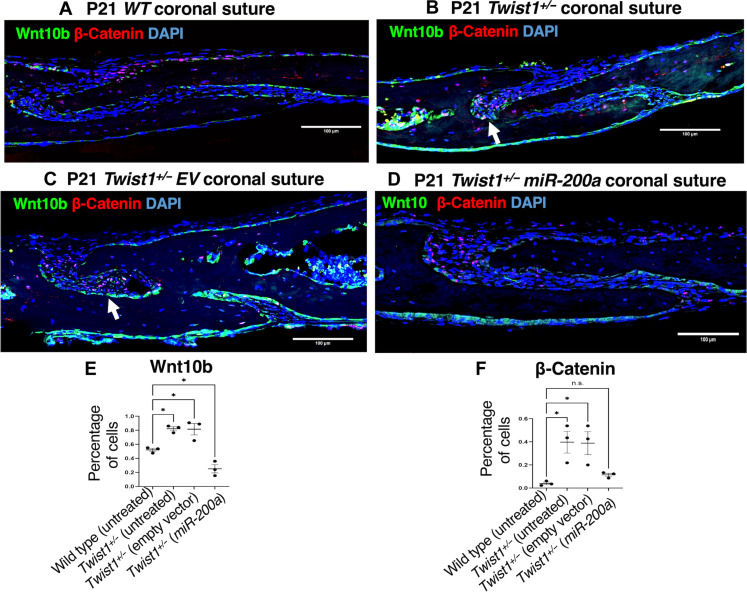
Wnt10b- and β-catenin–positive cells are increased in the *Twist1^+/−^* coronal sutures and decreased after *miR-200a* treatment. (**A**) Wnt10b and β-catenin IF expression in P21 WT mouse sagittal sections of the coronal suture. Wnt10b (green staining) and β-catenin (red staining) are expressed in suture cells. (**B**) Wnt10b and β-catenin IF expression in P21 *Twist1^+/−^* heterozygous mice, sagittal sections of the coronal suture. (**C**) Wnt10b and β-catenin IF expression in P21 *Twist1^+/−^* heterozygous mice treated with EV nanoparticles, sagittal sections of the coronal suture. (**D**) Wnt10b and β-catenin IF expression in P21 *Twist1^+/−^* heterozygous mice treated with *miR-200a* nanoparticles, sagittal sections of the coronal suture. (**E** and **F**) Quantitation of Wnt10b and β-catenin expression levels in the mouse sagittal sections (*n* = 3, three mice were analyzed for Wnt10b and β-catenin expression). *P < 0.05.

Wnt10b and β-catenin are part of the Wnt canonical signaling pathway associated with bone formation (*54*–*57*). Coronal sutures from P21 WT mice show Wnt10b and β-catenin expression in a subset of suture cells lining the surface of parietal and frontal bone and the osteogenic front (Fig. 8A). In both the P21 *Twist1^+/−^* untreated and EV-treated mice, there was an increase in Wnt10b and β-catenin at the osteogenic fronts (see arrow, Fig. 8, B and C). In P21 *Twist1^+/−^* mice after *miR-200a* treatment, both Wnt10b and β-catenin are decreased in the suture cells and osteogenic front (Fig. 8D). Quantitation of Wnt10b- and β-catenin–positive cells demonstrates an increase in *Twist1^+/−^* mice, compared to that in WT mice (Fig. 8, E and F). As both factors contribute to bone formation, their increased expression in *Twist1^+/−^* mice may facilitate premature suture fusion. In contrast, their decreased expression in *miR-200a*–treated *Twist1^+/−^* mice suggests that *miR-200a* regulates bone formation through the Wnt pathway (Fig. 8, E and F). In addition, we overexpressed *miR-200a* in bone marrow stem cells (BMSCs) and found that endogenous bone regulatory factors—*BGLAP*, *TGFB1*, *TGFBR1*, and *WNT5A—*were all decreased compared to that in EV-transfected cells (fig. S9).

### A single dose of PEG-peptide *miR-200a* nanoparticles has long-term effects

**Fig. 9.**
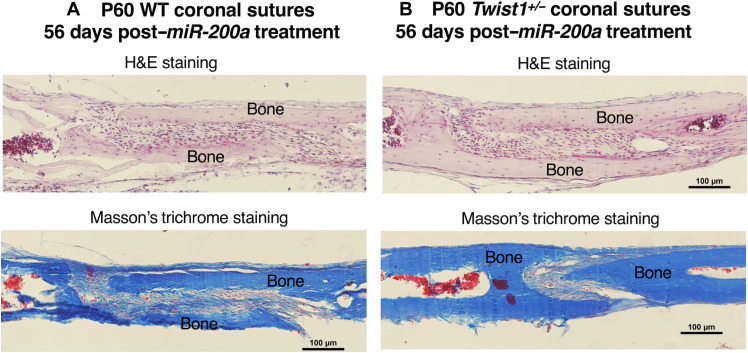
Long-term effect of a single *miR-200a* application to prevent premature suture fusion. (**A**) P60 WT coronal sutures were analyzed by H&E staining and Masson’s trichrome staining to determine whether *miR-200a* expression resulted in abnormal bone formation, fibrosis, or other anomalies 56 days post–*miR-200a* treatment. (**B**) P60 Twist1^+/−^ coronal sutures are shown 56 days post–miR-200a treatment. H&E staining shows normal bone structure and suture cells. Masson’s trichrome staining shows bone (blue) and unmineralized tissue including fibrosis and cells (red). Note: Red blood cells in the bone marrow stain red.

To determine long-term efficacy of *miR-200a* nanoparticle treatments, coronal sutures were harvested and analyzed after 56 days posttreatment. The treated *Twist1^+/−^* mouse sutures remained patent with no suture fusion 56 days after the initial treatment (Fig. 9, A and B). Furthermore, we did not observe abnormal bone growth or fibrosis in these mice using trichrome staining (Fig. 9, A and B). These results demonstrate a high level of efficacy and regeneration through one application of plasmid DNA expressing *pre-miR-200a* in PEG-peptide nanoparticles.

## DISCUSSION

Management of craniosynostosis requires multidisciplinary care teams that include pediatric neurologists, geneticists, plastic surgeons, neurosurgeons, and other specialists ideally in a tertiary healthcare center. During the first few years of life, treatment is mainly surgical intervention to relieve the fused suture/s. The goal is to reduce the risk of increased ICP, improve the head shape, and allow for normal brain development (*2*, *58*). Commonly performed surgical techniques include fronto-orbital advancement, open cranial vault remodeling, extended strip craniectomy, spring-assisted cranial expansion, and cranial vault distraction. Although techniques for initial cranial vault expansion and reshaping depend on the location and extent of deformity, variability in surgical practice patterns and surgeon experience has been reported in a recent national survey of craniofacial surgeons in the United States (*6*). Morbidity and complication rates also vary widely across different centers, ranging from 10 to 39% (*7*–*11*). There is a need for less invasive treatments for craniosynostosis.

We have been working on microRNA therapeutics for many years, and, through our bioinformatics analyses and mouse models, we determined that *miR-200a* was an option for treating suture fusion. We generated a *PMIS–miR-200a* mouse model that shows fusion of the coronal sutures by *miR-200a* inhibition. *PMIS–miR-200a* has also been used as a therapeutic to rapidly and efficiently regenerate bone (*59*). Therefore, if inhibition of *miR-200a* causes suture fusion and bone regeneration, then we reasoned that *miR-200a* overexpression would inhibit suture fusion in a mouse model for craniosynostosis. The ability to use gene therapy in a genetically altered cell/tissue environment (*Twist1^+/−^* mice) demonstrates the efficacy of these experiments.

To achieve efficient delivery of plasmid DNA expressing a pre-microRNA in vivo, we developed a PEGylated-peptide nanoparticle capable of packaging the plasmid DNA in a small 95-nm particle with a net positive charge. However, the composition of the PEGylated-peptide resists protein binding and thus allows it to enter the cell more efficiently. The formulation that we use is designed for in vivo uptake, release of the plasmid, and expression of *pre-miR-200a*, which is processed in the cell to a mature microRNA. This unique nanoparticle adds stability to the plasmid to allow for efficient expression for longer time periods. We demonstrate that suture cells take up the plasmid DNA and express GFP from the construct as proof of principle. PEGylation has been used to improve nanoparticle drug and gene delivery in many cells and tissues (*60*, *61*). Several PEGylated protein therapeutics have been approved for treatment of diseases (*61*). PEGylated nanoparticles were designed for systemic delivery as they increase circulation time and stability. The gene delivery polymer polyethylenimine (PEI) (*62*) has been replaced with a short, chemically optimized, nontoxic, and PEGylated (5-kDa PEG) polyacridine peptide (*36*, *37*, *63*, *64*). Polyacridine PEG-peptides bind tightly to plasmid DNA through poly-intercalation of Lys (Acr) residues and ionic binding of Lys residues. PEGylated polyacridine peptide DNA nanoparticles have proven to be versatile delivery agents that have a long circulatory half-life when dosed intravenously in mice and remain transfection competent for up to four hours in the circulation (*36*, *37*). Because of their remarkable in vivo stability and uniform PEG stealthing, these nanoparticles resist protein binding (*38*) and are fully serum compatible (*36*, *37*), and thereby these molecules remain functional when injected specifically to sutures. DNA nanoparticles having a disulfide-linked PEG undergo reduction of the disulfide bond by cell surface thiol reductases (*65*). The exposure of nanoparticle-positive charge at the cell surface facilitates transfection by nonspecific pinocytosis.

Another important discovery was using double-banded CsCl-prepared plasmid DNA for the nanoparticle formulation. The CsCl DNA reduced the nanoparticle size from 125 to 95 nm compared to commercially available column kit preparations. The CsCl plasmid DNA is devoid of proteins and RNA that bind the DNA and add to its size and modulate the charge of the DNA.

With the knowledge that our PEGylated-peptide DNA *miR-200a* nanoparticles can deliver the plasmid DNA to suture cells, we tested their effect to reverse suture fusion in *Twist1^+/−^* mice. A previous study used the *Twist1^+/−^* mice as a model to regenerate the coronal suture using a biodegradable material combined with mesenchymal stem cells (*52*). In that study, *Twist1^+/−^* mice with fused sutures were given a strip craniectomy, and the mesenchymal cells in a matrix were applied to the open suture. The treated *Twist1^+/−^* mice regenerated the suture and restored skull dysmorphology and neurocognitive dysfunctions (*52*). Our approach was to use noninvasive treatments before suture fusion in the *Twist1^+/−^* mice. Because craniosynostosis is only recognized after sutures begin to fuse in humans, most treatments require invasive surgical applications as we discussed previously. We treated eight littermates of *Twist1^+/−^* neonates to ensure that our treatments were effective. While we found suture fusion in all untreated mice, there were regions of the coronal suture that were not fused. These in-depth analyses demonstrate that craniosynostosis in the *Twist1^+/−^* mice can occur with regions of the suture unfused or in the process of fusing. In humans, partial synostosis of cranial sutures occurs and is sufficient to cause craniosynostosis (*66*). In studies of craniofacial shape variation in *Twist1^+/−^* mice, fusion at one or more regions of the coronal suture affected craniofacial shape (*67*). A recent study on coronal synostosis of the *Twist1^+/−^* reported partial fusion of the sutures (*68*). This is consistent with our studies showing a varying degree of coronal suture fusion in the *Twist1^+/−^* mice at P21. However, *miR-200a* treatments completely inhibited any suture fusion in all *Twist1^+/−^* mice.

To understand potential mechanisms for the action of *miR-200a* in suture patency, the thickness of the periosteum and dura mater was measured in the treated and untreated *Twist1^+/−^* mice. The periosteum and dura contain suture stem cells that maintain suture patency (*49*, *52*). It was previously shown that, using mesenchymal cells to regenerate the suture in *Twist1^+/−^* mice, progenitor cells from the dura contribute to suture patency (*52*). Our data demonstrate a specific thickening of the periosteum layer after *miR-200a* injection. The periosteum layer is populated by several cell layers not observed in WT, *Twist1^+/−^* EV–treated, or untreated mice. We speculate that the periosteum layer contributes to suture cells allowing for suture patency.

To identify cell populations in the suture mesenchyme, we stained for four well-known markers of suture cells. Because Twist1 is required for suture patency and regulation of Runx2 function to inhibit bone formation, we assayed for their expression in WT and *Twist1^+/−^* untreated and treated mice. As expected, Twist1 expression was decreased in the *Twist1^+/−^* untreated and treated mice compared to that in WT mice. However, Runx2 expression was not significantly affected by decreased *Twist1* expression or *miR-200a* treatments. These results suggest that the *miR-200a* mechanisms did not involve either of these two factors.

Gli1 and Six2 expression were both decreased in the *Twist1^+/−^* mouse coronal sutures at P21, compared to that in WT. While Gli1^+^ cells appear to be independent of Twist1 at later stages of suture development, it is not clear that these cells are suture stem cells (*47*, *49*, *52*). We observe a decrease in Gli1^+^ cells in *Twist1^+/−^* mice; however, this could result from decreased overall cell populations due to suture fusion. Gli1^+^ cells are restored to WT levels after *miR-200a* treatments. Six2^+^ cells have been shown to be suture progenitor cells and are a major component of the developing suture (*47*, *48*). An interesting discovery is that some cells in the suture appear to coexpress both Gli1 and Six2, but there are cell niches that only express one or the other. The dura mater cells appear to coexpress both factors. We speculate that these cells represent the heterologous nature of the suture cells.

### The role for *miR-200a* in suture biology

There are several reports of *miR-200a* as a negative regulator of osteogenesis [see review, (*69*)]. We demonstrate that *miR-200a* inhibition causes craniosynostosis in the *PMIS–miR-200a* mouse model. *miR-200a* does not directly regulate Twist1, Runx2, or Gli1, but it does modulate Smad and Wnt signaling (*31*, *69*). The decreased Wnt10b and β-catenin expression after *miR-200a* treatment in the suture cells and osteogenic front supports a role for *miR-200a* inhibiting bone formation. We have previously shown that β-catenin is a direct target of *miR-200a*; however, it is not known whether *Wnt10b* is a direct or indirect target of *miR-200a* (*30*). These data demonstrate a direct role for *miR-200a* inhibiting the Wnt canonical pathway. *WNT5a* is decreased in BMSCs transfected with *miR-200a*, suggesting that *miR-200a* may also target the Wnt noncanonical pathway. Other factors such as *BGLAP*, *TGFB1*, and *TGFBR1* were also decreased in the transfected BMSCs. These results indicate that *miR-200a* may be regulating several bone forming pathways.

We have previously shown a role for *miR-200a* in reprogramming mesenchymal cells (*30*). *Zeb2*, *vimentin*, *ITGB1*, and *Col1A2* were increased after *miR-200a* expression in MDPC cells, whereas *Lef-1* and β*-catenin* were decreased in these cells (*30*). Recently, it was suggested that Wnt signaling up-regulation promotes osteogenic differentiation of suture mesenchymal cells and contributes to craniosynostosis (*52*). *miR-200a* directly targets β-catenin to regulate Wnt signaling and reduces cyclin D2 expression (*30*). Therefore, our results suggest a role for *miR-200a* in suture patency through the regulation of Wnt and Smad signaling to inhibit osteogenesis.

We report a simple noninvasive method to treat premature suture fusion by injection of nanoparticles containing a plasmid DNA expressing a *pre-miR-200a*. Because most cases of craniosynostosis are not diagnosed until after birth and suture fusion has begun, our treatment would require a strip ectocraniectomy followed by an injection of the *miR-200a* nanoparticle. However, this would be a relatively less invasive procedure and may only require one application to retain suture patency. If newborns were identified with craniosynostosis before suture fusion, then this approach would be completely free of surgical approaches.

## MATERIALS AND METHODS

### Animals

Mice are maintained in the animal facility of the University of Iowa. All experiments were approved by the Institutional Animal Care and Use Committee of the University of Iowa. The construction of *PMIS* inhibitor mice (C57BL/6) were previously described (*24*). The *Twist1* general KO mouse strain was obtained from the Jackson Laboratory (Jackson strain no. 002221). The *Mesp1^Cre^* and *Twist1^Flox/Flox^* mice were both gifts from R. Maxson (University of Southern California). The conditional *Twist1* cKO mice were generated by crossing the *Mesp1^Cre^* to the *Twist1^Flox/Flox^* mice. Subcutaneous scalp injections were performed on mice with nanoparticle PEG/DNA in the region just superior to the coronal suture; 10 to 15 μl were injected (0.06 nmol of PEG, DNA at 0.2 μg/μl) at P4; and mice were euthanized at P10, P15, or P21 for analysis of the efficacy and effectiveness of the treatment. Both male and female mice were analyzed in all experiments.

### Cell culture

BMSCs were purchased (American Type Culture Collection) and grown as recommended by the supplier. PEI transfections were used to transfect BMSCs with plasmid DNA overexpressing *miR-200a* or EV, and RNA was isolated from the cells using TRIzol reagent (Thermo Fisher Scientific) and subjected to quantitative polymerase chain reaction primers for *BGLAP*, *TGFB1*, *TGFBR1*, and *Wnt5a*. Each experiment was performed with three independent replicates. Primer sequences are listed in Table 1.

**Table 1.** Primer sequences for quantitative polymerase chain reaction.

Gene	Forward primer	Reverse primer
BGLAP	GCAATAAGGTAGTGAACAGACTCC	CCATAGATGCGTTTGTAGGCGG
TGFB1	TGATACGCCTGAGTGGCTGTCT	CACAAGAGCAGTGAGCGCTGAA
TGFBR1	TGCTCCAAACCACAGAGTAGGC	CCCAGAACACTAAGCCCATTGC
WNT5A	GGAACGAATCCACGCTAAGGGT	AGCACGTCTTGAGGCTACAGGA

### PEG-peptide DNA nanoparticles

A polyacridine peptide of sequence C(-Acr-K_4_)_3_-Acr-K, where Acr is a Lys residue with its ε-amine modified with acridine, was synthesized using methods previously described (*38*). Briefly, the polyacridine peptide obtained from solid-phase peptide synthesis was purified by reverse-phase high-performance liquid chromatography (RP-HPLC). The crude peptide was dissolved in 0.1% trifluoroacetic acid and eluted at 10 ml/min with an acetonitrile gradient (15 to 65% over 30 min) while collecting the product peak detected by absorbance 409 nm (Acr, ε = 9266 M^−1^ cm^−1^). Purified polyacridine peptide was reacted with tris(2-carboxyethyl)phosphine hydrochloride to reduce the N-terminal Cys residue to direct its reaction with PEG. The reduced polyacridine peptide was reacted with ortho-pyridyldisulfide PEG (OPSS-PEG_5kDa_, Sigma-Aldrich) in Hepes buffer (100 mM, pH 7.0) to generate the desired disulfide-linked PEG-peptide. The PEG-peptide was purified to homogeneity by RP-HPLC and then concentrated by freeze drying. The counterion was exchanged to acetate by dissolving the PEG-peptide in 0.1% acetic acid and freeze drying. The purified PEG-peptide was dissolved in water and characterized by mass spectroscopy (matrix-assisted laser desorption/ionization–time-of-flight mass spectrometry), which resulted in an average mass of 7820.857 mass/charge ratio. Plasmid DNA was purified by alkaline lysis and CsCl banding to provide highly purified DNA. PEG-peptide DNA nanoparticles were prepared by combining 0.3 nmol of PEG-peptide with 1 μg of DNA in water resulting in the spontaneous formation of DNA nanoparticles (40 μg/ml) of ~100 to 150 nm in diameter as determine by dynamic light scattering (*38*).

### Tissue processing and IF

After fixation in 4% paraformaldehyde, the top of mouse skulls was decalcified in 11% EDTA for 4 to 5 days, dehydrated and put into paraffin blocks, sectioned at 7 μm, and stained with hematoxylin and eosin (H&E) or used for IF stain as described earlier (*70*). To evaluate nanoparticle uptake efficiency, IF staining was performed for GFP, and, for markers of mesenchymal cells, Twist1 (Thermo Fisher), Gli1 (Santa Cruz Biotechnology Inc.), Runx2 (Cell Signaling Inc.), Six2 (Proteintech Inc.), Wnt10b (Abcam), and β-catenin (Developmental Studies Hybridoma Bank) antibodies were used. Sections were incubated overnight with primary antibody then further washed with phosphate-buffered saline (PBS) and incubated with secondary antibody, goat anti-rabbit or goat anti-mouse Alexa Fluor 488 or Alexa Fluor 555 (Life Technologies, Camarillo, CA, USA), for 60 min at room temperature, followed by incubation with DAPI for 25 min. Cells or sections were imaged using a fluorescence Nikon Eclipse Ts2 microscope (Eclipse Ts2; Nikon Instruments Inc., Melville, NY, USA) or the Leica 680 confocal microscope. ImageJ was used to quantitate the fluorescent intensity with DAPI used as a control.

#### Imaging and micro–computed tomography

Mouse skulls from three experimental and control animals were scanned with a Siemens Inveon Micro-CT/PET scanner using 60 kVp and 500 mA with a voxel size of 30 μm. Reconstructed images were imported using Osirx DICOM software. Mouse heads were prepared by overnight fixation at 4°C, followed by storage in 70% ethanol. Scans directed across the anterior-posterior plane produced two-dimensional images that were matched between animals using topology markers such as the molar. ImageJ (Fiji) software was used to measure the thicknesses of both the periosteum and dura mater layers. A representative H&E-stained section was chosen for each animal, and 10 technical replicates were obtained, where the layers were measured at 10 different arbitrary locations across one section. These 10 measurements were then averaged out to represent each individual animal, and this protocol was repeated for all eight biological replicates of each experimental group. ImageJ (Fiji) software was used to analyze all images obtained from IF. A region within the coronal suture was analyzed, bounded by an arbitrary 100 μm–by–100 μm box. Within the box, cells were counted as a ratio of cells that stained positively for a marker of interest (*Twist1*, *Gli1*, *Six2*, and *Runx2*) over the total number of cells that were DAPI positive. These ratios were then recorded as “percentage of cells” values, and this process was repeated for all three or four biological replicates of each experimental group.

#### Statistics

Descriptive statistics were conducted, and one-way analysis of variance (ANOVA), followed by the post hoc Tukey-Kramer test, was used to detect the difference among the experimental groups. Statistical analyses were performed using GraphPad Prism 10 (GraphPad Software Inc., USA), and *P* values were calculated using two tailed, unpaired *t* tests using at least three biological replicates for each group. For Fig. 5 (B and D), an ordinary one-way ANOVA was applied, using the Dunn-Šidák correction to assess the thicknesses of both the periosteum and dura mater. For Figs. 6 and 7, an ordinary one-way ANOVA was applied, using Dunnett’s multiple comparisons test as a correction. For fig. S7A, multiple Mann-Whitney tests were applied, because the weights of all experimental groups were recorded without regard for sex; therefore, the sample population was not assumed follow normal distribution. For fig. S7B, a Kruskal-Wallis test was applied to compare the weights of the mutants against each other. Error bars are presented as ±SE of the mean in every figure. *P < 0.05, **P < 0.01, ***P < 0.001, and ****P < 0.0001.
