# Supplementary material for: Inhibition of craniosynostosis and premature suture fusion in Twist1 mutant mice with RNA nanoparticle gene therapy
Source: Sci Adv. 2025 Aug 22;11(34):eadx9763. doi: 10.1126/sciadv.adx9763 (PMC12372882; doi:10.1126/sciadv.adx9763)
Supplement: Supplementary file 1 — Figs. S1 to S9 [file sciadv.adx9763_sm.pdf]

Science Advances
AAAS

Supplementary Materials for

Inhibition of craniosynostosis and premature suture fusion in *Twist1* mutant mice with RNA nanoparticle gene therapy

Samuel Swearson *et al.*

Corresponding author: Brad A. Amendt, brad-amendt@uiowa.edu

Sci. Adv. **11**, eadx9763 (2025)
DOI: 10.1126/sciadv.adx9763

This PDF file includes:

Figs. S1 to S9

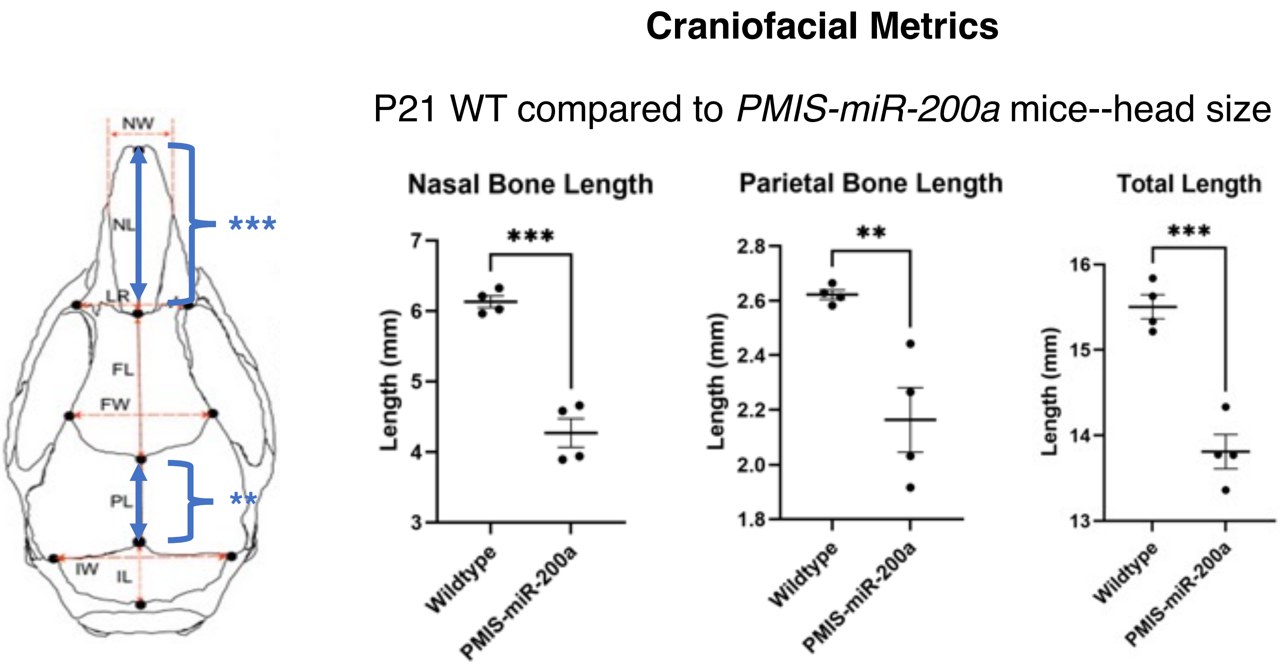

Fig. S1. Craniofacial measurements of *PMIS-miR-200a* mice. We used known landmarks to measure the size differences in the P21 *PMIS-miR-200a* heads compared to WT. Significant differences in head size were found in the nasal bone length ($p< 0.005$) and parietal bone length, which affected total length ($p<0.01$)($N=4$).

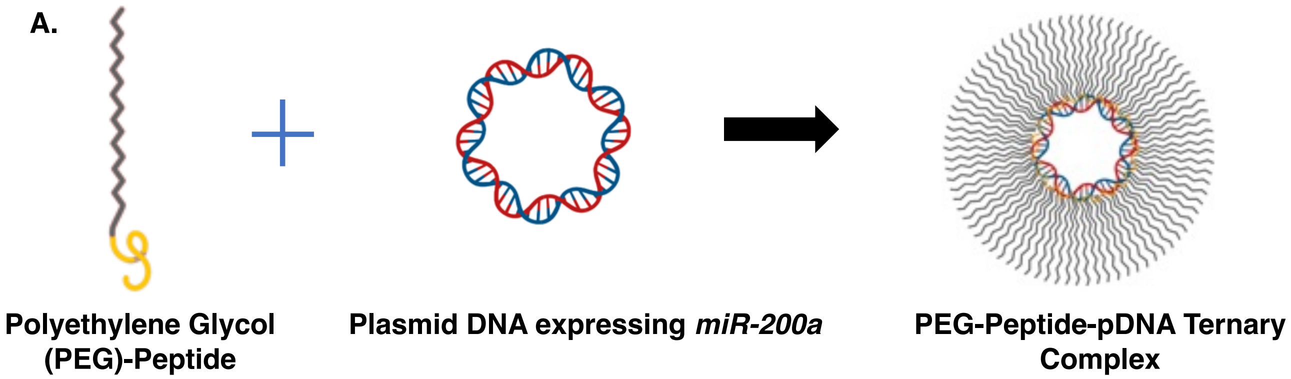

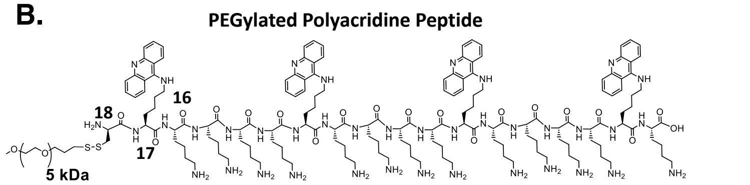

	PEG linkage	Linkage	Sequence 17 & 16	Protein Binding
**PEG-peptide 1**	Disulfide	Reducible	Lys(Acr)-Lys	Resists
**PEG-peptide 2**	Maleimide	Stable	Lys(Acr)-Lys	Resists
**PEG-peptide 3**	Disulfide	Reducible	Lys-Lys(Acr)	Supports
**PEG-peptide 4**	Maleimide	Stable	Lys-Lys(Acr)	Supports

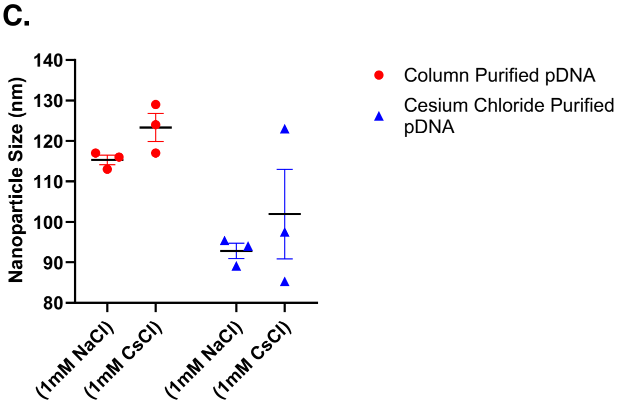

Dynamic light scattering (DLS) measurements showed the average CsCL plasmid DNA nanoparticle size was 95nm (blue triangles) used in experiments.

Fig. S2. PEGylated peptide nanoparticle formulation and plasmid DNA nanoparticles. A) Schematic of plasmid DNA PEG-peptide nanoparticle complex formation. Created in BioRender. Swearson, S. (2025) https://BioRender.com/aaqt4yg. **B)** Different PEG-peptides used in determining the most efficient in vivo nanoparticle delivery system. **C)** Measurements of nanoparticle size using dynamic light scattering and how CsCl purified plasmid DNA produces smaller nanoparticles than commercially available column purification techniques. Plasmid DNA purified by commercial reagents or CsCL double-banded were resuspended in either NaCL or CsCL prior to nanoparticle formation.

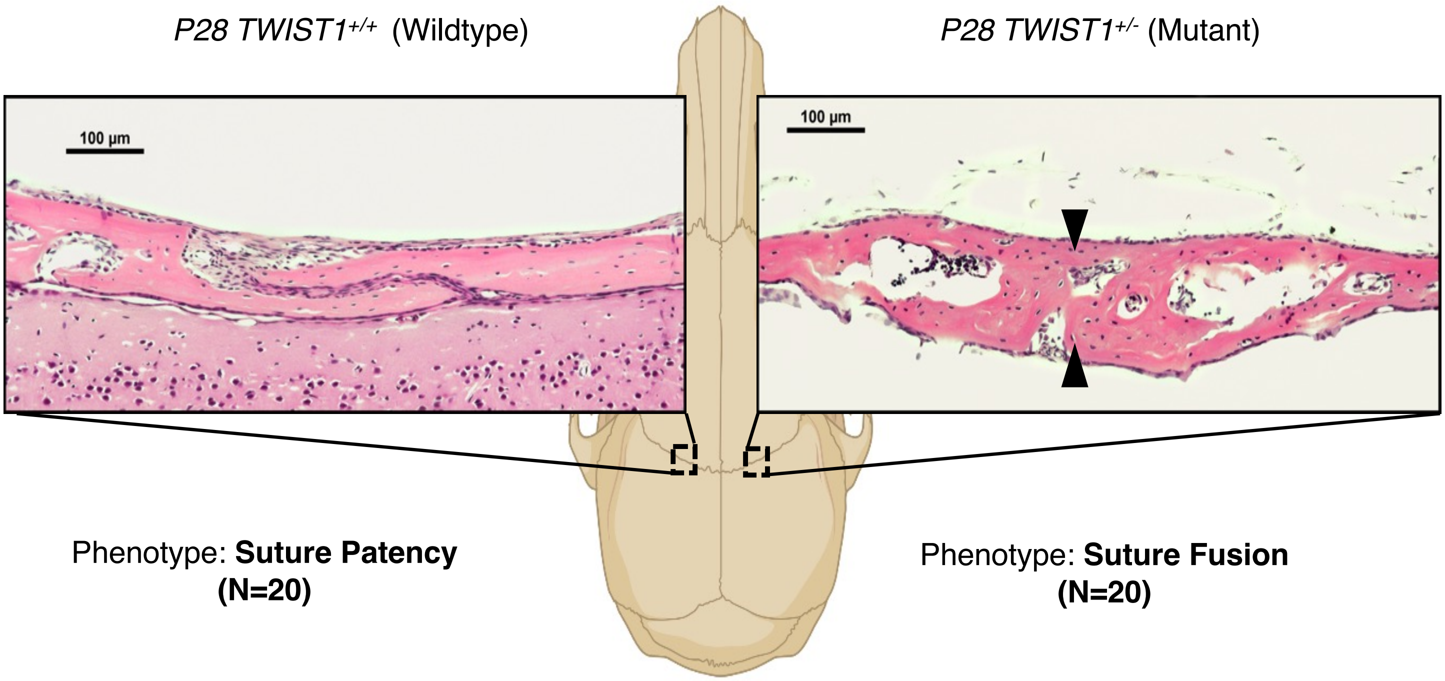

Fig. S3. Diagram and description of suture sections. Sutures were identified by sagittal sectioning of the coronal sutures in P28 WT (open sutures) or P28 *Twist1*$^{+/-}$ heterozygous mice (closed or fused sutures). Multiple mice were analyzed for suture patency in the study (N=20).

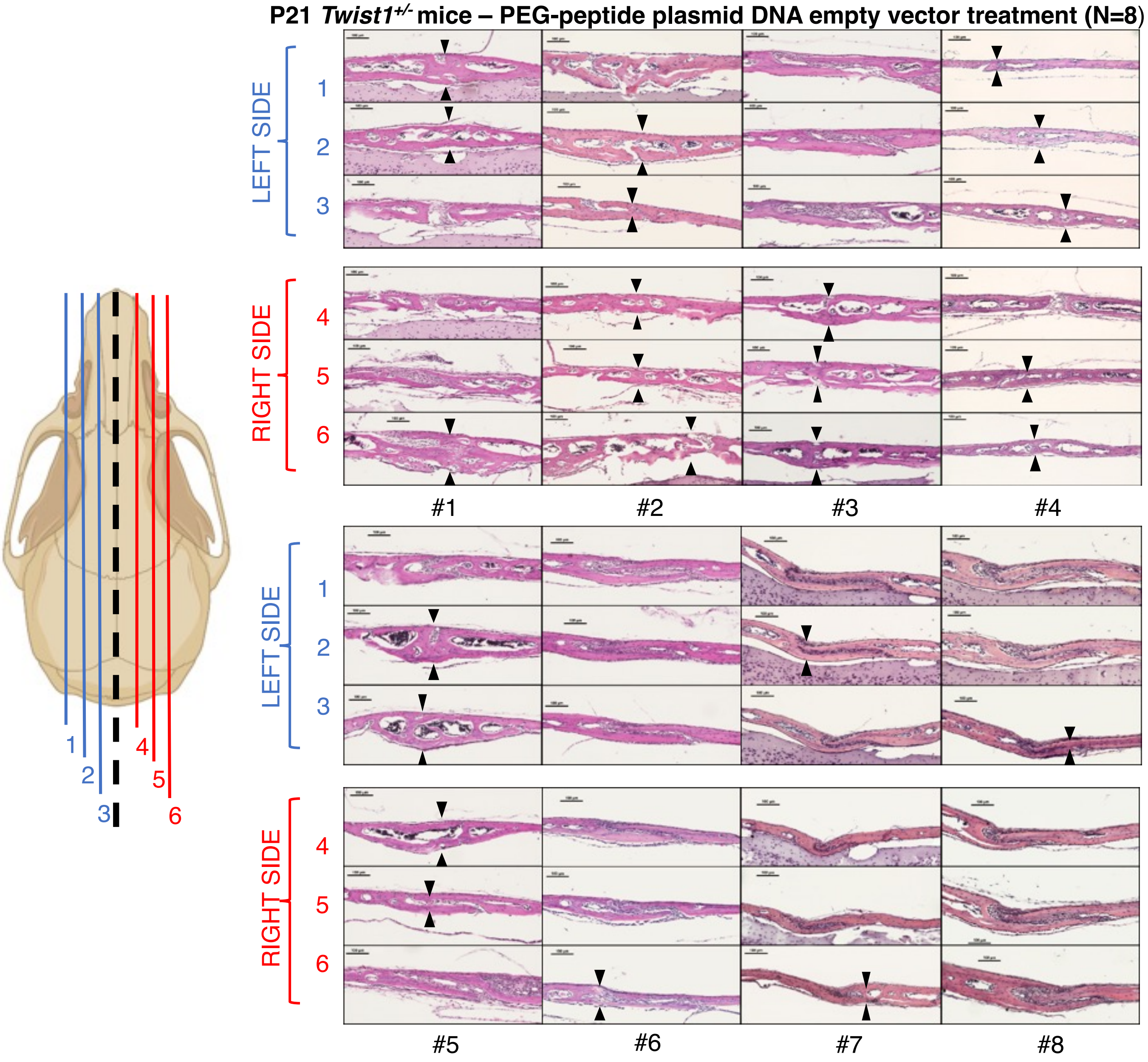

Fig. S4. Serial sections of P21 *Twist1*$^{+/-}$ mice treated with empty vector nanoparticles. While every *Twist1*$^{+/-}$ mouse treated with EV nanoparticles had fused sutures (fusion at any place in the coronal suture was considered fused), the 8 *Twist1*$^{+/-}$ mice analyzed in this figure show varying amounts of suture fusion (arrowheads). Thus, these mice do not show complete suture fusion at P21, which is why multiple mice were analyzed after treatments.

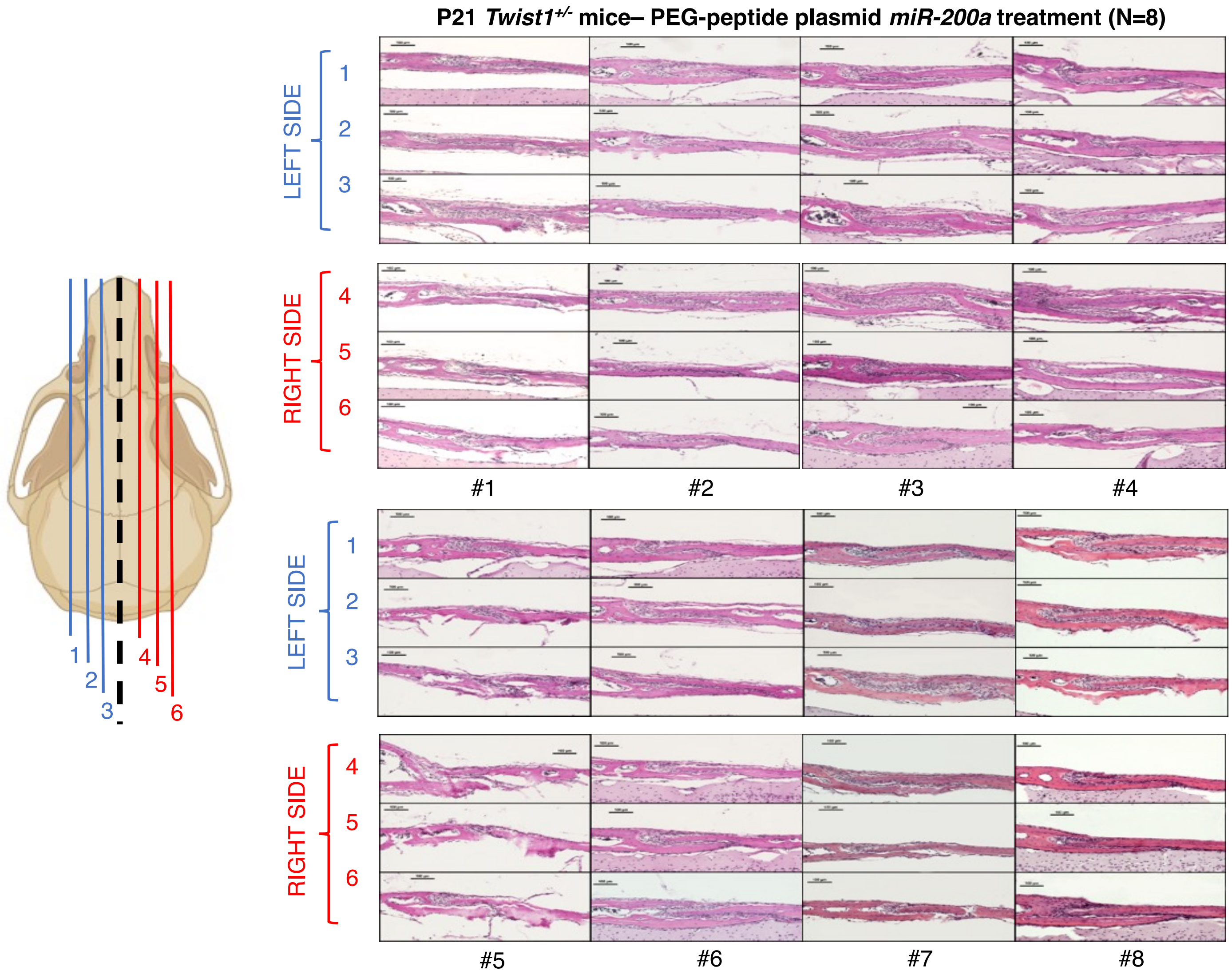

Fig. S5. All P21 *Twist1*$^{+/-}$ mice treated with *miR-200a* show suture patency. All 8 P21 *Twist1*$^{+/-}$ treated with PEG-peptide plasmid DNA expressing *miR-200a* showed coronal suture patency. Treatment with *miR-200a* inhibited suture fusion in all *Twist1*$^{+/-}$ mice.

Protocol: At post-natal day 5 (P5) 5 ug of plasmid DNA nanoparticles injected under the scalp of mice prior to coronal suture fusion in the *Mesp1*^{Cre}/*Twist1*$^{+/F}$ mice. Mice were analyzed at P21 to analyze suture formation

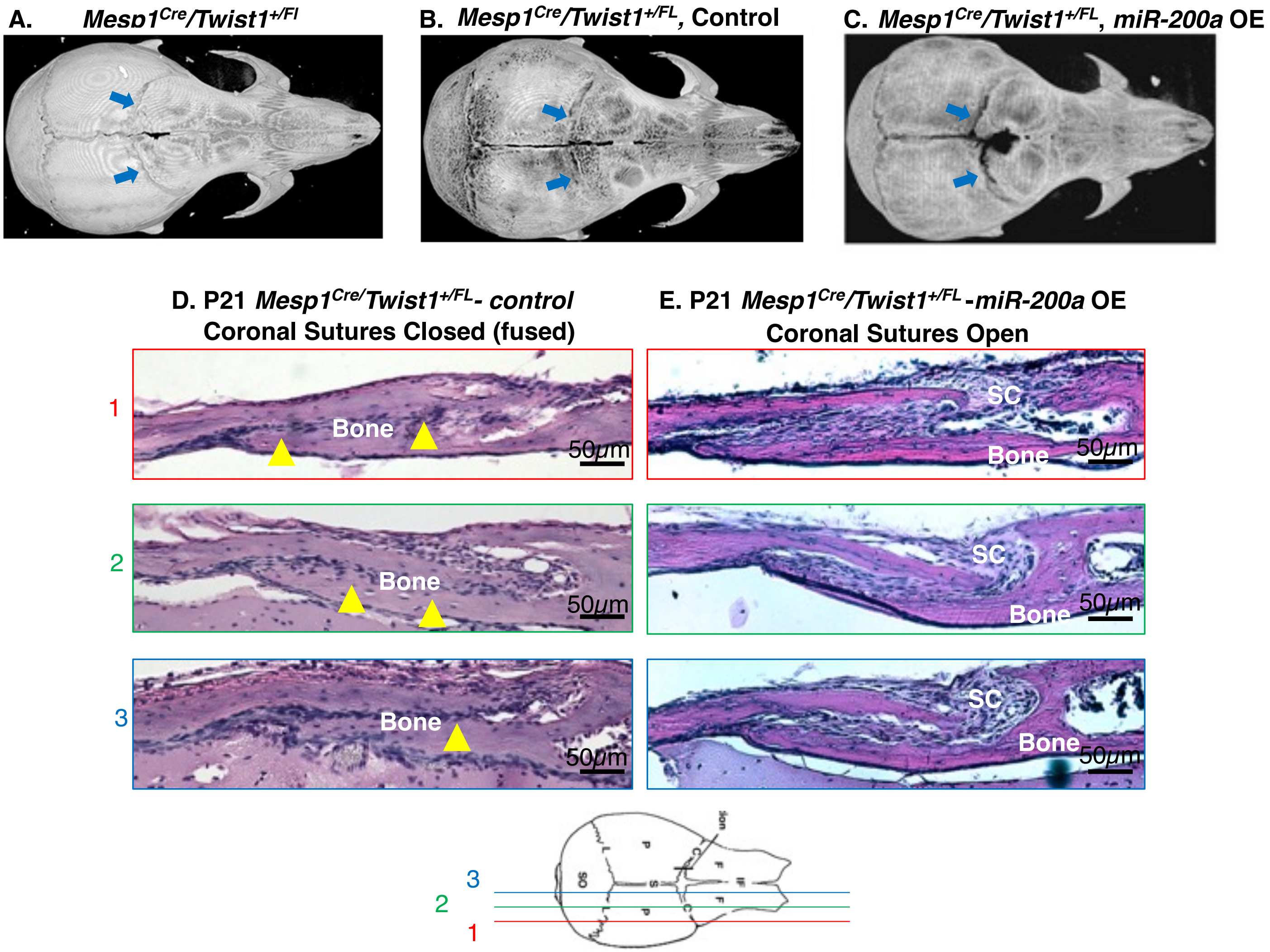

Fig. S6. Conditional heterozygous *Mesp1*^{Cre}*/Twist1*$^{+/FL}$ mice treated with *miR-200a* show suture patency.

A) µCT image of P21 *Mesp1*^{Cre}*/Twist1*$^{+/FL}$ mice showing fused coronal sutures (blue arrows). **B)** µCT image of P21 *Mesp1*^{Cre}*/Twist1*$^{+/FL}$ mice treated with empty vector (control) showing fused coronal sutures (blue arrows). **C)** µCT image of P21 *Mesp1*^{Cre}*/Twist1*$^{+/FL}$ mice treated with PEG-peptide plasmid DNA expressing *miR-200a* showing open coronal sutures (blue arrows). **D)** H&E stained sagittal sections of P21 *Mesp1*^{Cre}*/Twist1*$^{+/FL}$ mice treated with empty vector (control) showing fused coronal sutures (yellow arrowheads). **E)** H&E stained sagittal sections of P21 *Mesp1*^{Cre}*/Twist1*$^{+/FL}$ mice treated with PEG-peptide plasmid DNA expressing *miR-200a* showing open (patent) coronal sutures. SC, suture cells; sections 50µm.

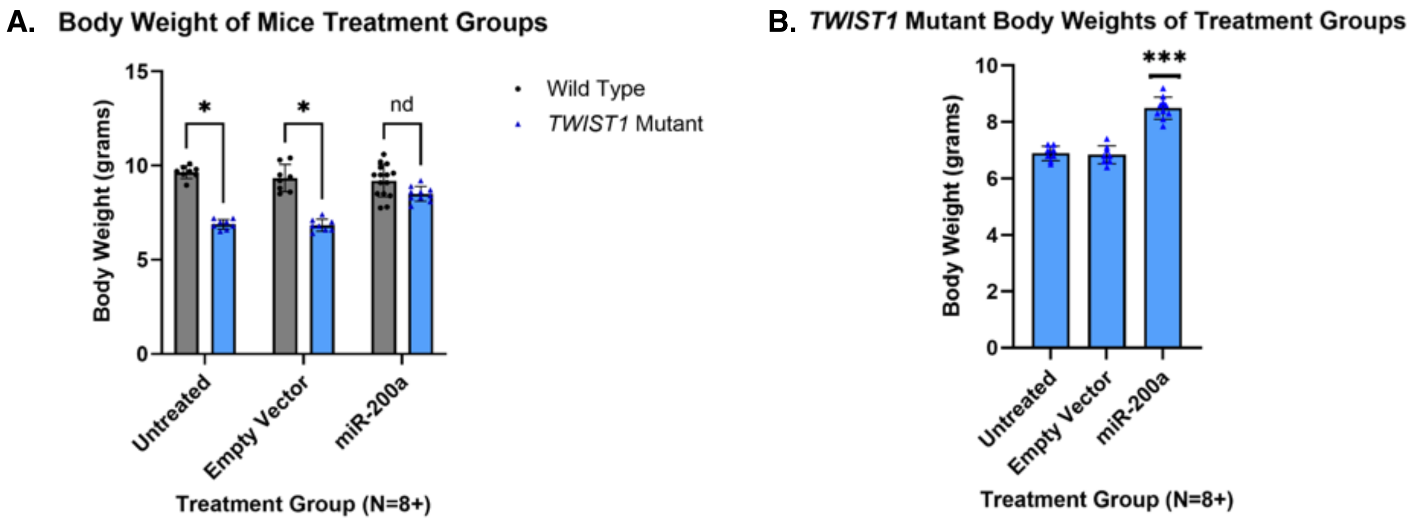

Fig. S7. *Twist1*$^{+/-}$ mice treated with *miR-200a* grow similar to WT mice. **A)** *Twist1*$^{+/-}$ mice at P21 weigh less than their WT counterparts. *Twist1*$^{+/-}$ mice (P21) treated with empty vector weigh less than WT mice (P21). *Twist1*$^{+/-}$ mice (P21) treated with PEG-peptide plasmid DNA expressing *miR-200a* regain weight and are not significantly smaller than WT mice (P21). **B)** *Twist1*$^{+/-}$ untreated, empty vector treated and *miR-200a* treated body weights show a significant increase in the body weight of the *miR-200a* treated mice.

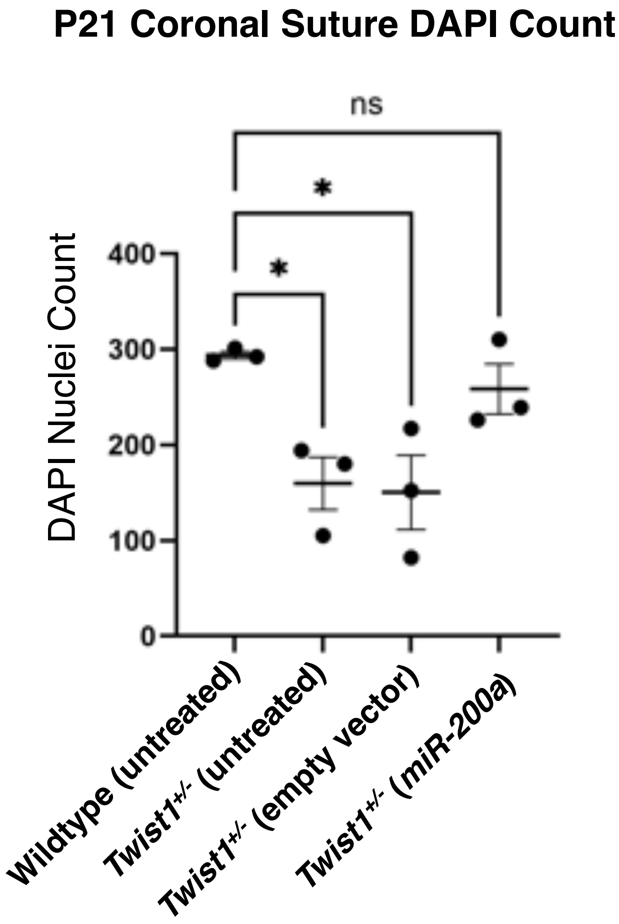

Fig. S8. Suture cell numbers are restored in the *Twist1*$^{+/-}$ mice treated with *miR-200a*. Suture cell numbers (DAPI counts) were calculated from coronal suture sections of WT, *Twist1*$^{+/-}$, *Twist1*$^{+/-}$ EV (empty vector) and *miR-200a* treated mice. N=3, *p<0.05. *Twist1*$^{+/-}$ mice treated with *miR-200a* regained suture cells, similar to WT.

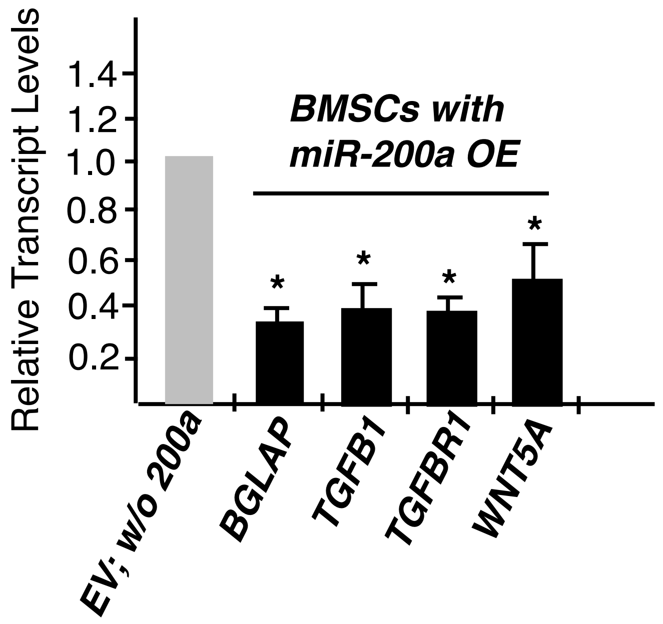

Fig. S9. ***miR-200a*** **regulates critical genes involved in bone formation.** Bone Marrow Stem Cells (BMSCs) were transfected with a plasmid expressing *pre-miR-200a* or empty vector (EV). Cells were harvested and RNA was analyzed for gene expression by qPCR. *BGLAP, TGFB1, TGFBR1* and *WNT5A* transcripts were all decreased by *miR-200a* over expression. N=3, *p<0.05.
